# Spatial–Temporal Distribution of Megamouth Shark, *Megachasma pelagios*, Inferred from over 250 Individuals Recorded in the Three Oceans

**DOI:** 10.3390/ani11102947

**Published:** 2021-10-12

**Authors:** Chi-Ju Yu, Shoou-Jeng Joung, Hua-Hsun Hsu, Chia-Yen Lin, Tzu-Chi Hsieh, Kwang-Ming Liu, Atsuko Yamaguchi

**Affiliations:** 1 Department of Environmental Biology and Fisheries Science, National Taiwan Ocean University, Keelung 20224, Taiwan; wing13260@gmail.com (C.-J.Y.); skinomotohiko@gmail.com (C.-Y.L.); andy760327@gmail.com (T.-C.H.); 2 Graduate School of Fisheries Science and Environmental Studies, Nagasaki University, Nagasaki 852-8521, Japan; y-atsuko@nagasaki-u.ac.jp; 3 George Chen Shark Research Center, National Taiwan Ocean University, Keelung 20224, Taiwan; kmliu@mail.ntou.edu.tw; 4 Marine Studies Section, Center for Environment and Water, Research Institute, King Fahd University of Petroleum and Minerals, Dhahran 31261, Saudi Arabia; hsuhuahsun@yahoo.com.tw; 5 Institute of Marine Affairs and Resource Management, National Taiwan Ocean University, Keelung 20224, Taiwan; 6 Center of Excellence for the Oceans, National Taiwan Ocean University, Keelung 20224, Taiwan

**Keywords:** horizontal movement, vertical movement, elasmobranchs, sex segregation, western North Pacific, eastern Taiwan waters

## Abstract

**Simple Summary:**

In this study, we integrate *Megachasma pelagios* records from the three oceans, refine previous results, add more individual data, solve the problem of uncertain body size estimations, and provide additional information on the horizontal and vertical distributions. A checklist of over 250 *M. pelagios* is integrated in this study based on numerous public sources, published papers, personal communication, and unreleased information, especially the catch records from Taiwanese waters. The conversion equations among different length measurements are provided. In addition, the spatial–temporal movement of *M. pelagios* is inferred from the integrated data, and the results may provide important information on the vertical and geographic migration behavior of the mysterious species.

**Abstract:**

The megamouth shark (*Megachasma pelagios*) is one of the rarest shark species in the three oceans, and its biological and fishery information is still very limited. A total of 261 landing/stranding records were examined, including 132 females, 87 males, and 42 sex unknown individuals, to provide the most detailed information on global megamouth shark records, and the spatial–temporal distribution of *M. pelagios* was inferenced from these records. The vertical distribution of *M. pelagios* ranged 0–1203 m in depth, and immature individuals were mostly found in the waters shallower than 200 m. Mature individuals are not only able to dive deeper, but also move to higher latitude waters. The majority of *M. pelagios* are found in the western North Pacific Ocean (>5° N). The Indian and Atlantic Oceans are the potential nursery areas for this species, immature individuals are mainly found in Indonesia and Philippine waters. Large individuals tend to move towards higher latitude waters (>15° N) for foraging and growth from April to August. Sexual segregation of *M. pelagios* is found, females tend to move to higher latitude waters (>30° N) in the western North Pacific Ocean, but males may move across the North Pacific Ocean.

## 1. Introduction

The megamouth shark, *Megachasma pelagios* [1], is one of the most spectacular discoveries of a new shark species in the late 20th century [2]. The first record of this remarkable species was an individual caught accidentally from an entanglement in parachutes that had been deployed from a research vessel operated by the Naval Undersea Center, Kaneohe, Hawaii, on 15 November 1976 [1]. The specimen was retained and sent to the Bernice P. Bishop Museum, Honolulu, Hawaii, for further study. The specimen measured 446 cm (14.6 ft) total length (TL) and weighed 750 kg (1653 lbs.). The species was considered rare for decades since fewer than 50 individuals were recorded until 2010 [3]. However, it has become clear that this species is more common than previously thought in recent years. In fact, to date, new records extending its geographic range continue to surface [4,5,6,7,8].

*Megachasma pelagios* reaches a maximum length of at least 820 cm TL, with females maturing at nearly 600 cm TL and males at 425 to 450 cm TL [5,9]. Its size at birth based on the smallest known individuals has been estimated at approximately 170 cm TL [10,11,12]. Although its fecundity or reproductive mode is not clear, scientists suggest that it may be viviparous with oophagy due to its similarity to other lamniform sharks [9,13]. It appears to feed by swimming through dense aggregations of krill and shrimp [14,15]. This is primarily an oceanic species usually found offshore in very deep water from 0 to 1500 m deep, but may also occasionally occur over continental shelf waters at 5–40 m depth [9]. Due to the scarcity of biological and catch data, it has been categorized as of least concern on the red list by the IUCN [16].

On the other hand, a resource management strategy has been followed and *M. pelagios* retention has been prohibited in United States Pacific fisheries since 2004, but the rule was refined for scientific or educational use in 2015, indicating the lack of information regarding this species. Afterward, the Taiwan Fisheries Agency announced a ban fishing management measure on *M. pelagios* on 10 November 2020 for conservation purposes (the catching is forbidden; fisherman have to release the shark whether it is dead or alive); however, further effectiveness and study remain to be elucidated. Studies to date have included its morphology, movement, molecular biology, physiology, and chemical analysis [17,18,19,20,21,22,23,24,25].

Although *M. pelagios* was mainly recorded in the western North Pacific Ocean in previous studies, a recent study using molecular technology indicated low genetic diversity of this species in the Pacific Ocean, suggesting that it may have the ability to move across ocean basins [23]. Mature individuals are able to move to higher latitudes and may be segregated by sex, and gravid females may pup in warmer waters [3,6]. However, these studies were based on a small sample size, very few individuals between 250 and 400 cm TL were included, individuals were uncommon at lower latitudes from April to October in the Northern Hemisphere, and there was insufficient information on latitudinal distribution to reach any conclusion.

In addition, recent *M. pelagios* landing records were also found in different public sources or published papers, but these data are very scattered and incomplete and need to be integrated [7,8]. There were many unpublished data or unreleased information that needed to be included; for example, numerous individuals were recorded by the mandatory catch and report system of the Taiwan Fisheries Agency, and some individuals were recorded by the Japanese Society for Elasmobranch Studies in Japanese. Therefore, the present study aims to (1) integrate records of *M. pelagios* from the three oceans, (2) refine previous results and solve problems such as small sample sizes or uncertain body size estimations, and (3) provide additional information on the horizontal and vertical distributions of *M. pelagios*. It is hoped that the complete global landing data and spatial–temporal distribution of *M. pelagios* derived from these records can provide useful information for better understanding the ecology of this mysterious species.

## 2. Materials and Methods

### 2.1. Data Collection and Integration

The data used in this study were collected from published scientific articles, gray literature, online information, news, social network service (SNS) resources, private contact with research institutes, and interviews with fishermen and researchers. In addition, the following public websites were reviewed for *M. pelagios* records: Florida Museum [26], Sharkman’s World [27], Summary of Megamouth Sharks [28], Japanese Society for Elasmobranch Studies [29], and catch and report data from the Taiwan Fisheries Agency, Council of Agriculture [30]. To confirm the accuracy of these data, we cross-validated and checked each record from the above sources, including date, time, method (fishing gear, sighting, or stranded), location, operation depth, record country, length, sex, and maturity, if available. The data published in journals would be the most convincing, others (e.g., unpublished online resources and personal communication) were cross-checked from different sources. This information was used for further estimation of the spatial–temporal distribution of *M. pelagios*.

### 2.2. Bycatch in Taiwan Fisheries

**Figure 1:**
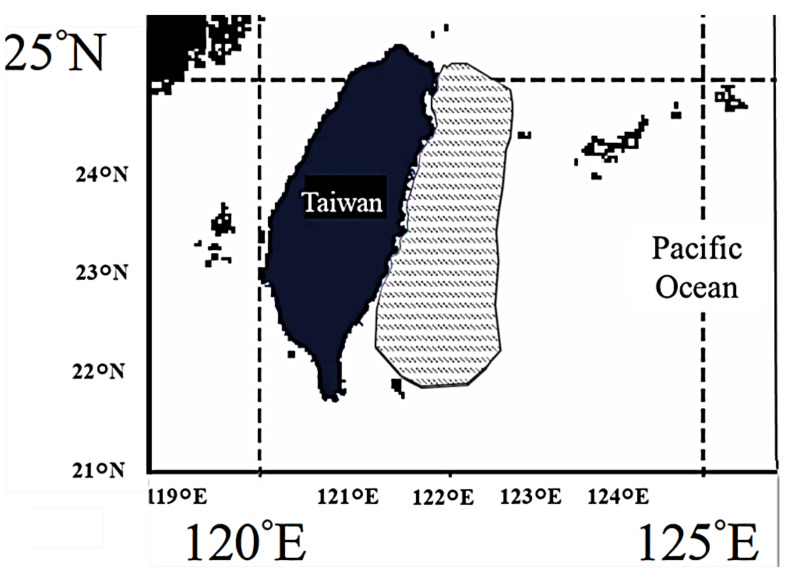
Fishing ground of *M. pelagios*, in the eastern Taiwan waters (shaded area is the operation area of large-mesh drift net fishery).

There has been a mandatory catch and report system for *M. pelagios* in Taiwan since 2013, and fishermen have to report their catch information to scientific institutions and the Fishery Agency when they catch megamouth sharks. Almost all *M. pelagios* records from Taiwan were from the bycatch of large-mesh (mesh size = 90 cm) drift net vessels, which operated in the eastern waters of Taiwan, targeting ocean sunfish (*Mola mola*), sharptail mola (*Masturus lanceolatus*), and Indo-Pacific sailfish (*Istiophorus platypterus*), especially during April–August (Figure 1). According to interviews with fishermen, the large-mesh drift net fishery operates primarily in the evening (18:00–24:00) with net deployment ranging from 10 to 140 m in depth and ~2000 m wide, soaking for 2–3 h. Only a few landed specimens were accidentally caught by trawl nets and longline vessels.

### 2.3. Meristic Measurement

Measurements were taken on total length (TL in cm), precaudal length (PCL), fork length (FL), body weight (BW in kg), mouth width (MW), 1st dorsal fin anterior margin, (D1A), 1st dorsal fin height (D1H), 1st dorsal fin base (D1B), pectoral fin anterior margin (P1A), and caudal fin dorsal margin (CDM) of those sharks landed at Taiwanese fish markets following the protocol described by Ebert et al. [9]. These data were used to develop conversion equations between different measurements and length–weight relationships.

### 2.4. Maturity Stage and Distribution

To understand the monthly horizontal distribution of *M. pelagios* in different life stages, the maturity stage was identified by macroscopic examination of reproductive organs if possible. Three maturity stages of *M. pelagios* were categorized as: immature, maturing, and mature. The maturity stage of the individuals not in Taiwanese waters was based on the descriptions of other resources. The maturity stage of *M. pelagios* in Taiwanese waters was determined by the following criteria: Stage I (immature): immature males and females have undeveloped gonads, testis, and ovaries were small or nondistinguishable, vas deferens and oviducts were small in diameter, clasper were uncalcified, and uteri were threadlike. Stage II (maturing): developing (transitional) reproductive organs were observed in males by clasper development (could be slightly rotated) and the presence or absence of semen, and inflating ovaries or uteri were observed in females. Stage III (mature): developed claspers (could be rotated), inflated testis, and semen were found in males; mating scars, inflated ovaries, and large uteri were found in females. The vertical distribution of *M. pelagios* at different sizes and time was plotted, and depth data included reliable catch depths or operation depths from fishermen and observers of NOAA (National Ocean and Atmospheric Administration) Fisheries.

### 2.5. Data Analysis

A linear regression analysis was used to describe relationships for TL-FL, TL-PCL, TL-MW, TL-D1A, TL-D1H, TL-D1B, TL-P1A, and TL-CDM. An allometric equation (BW = *a*TL*^b^*) was used to describe the relationship between BW and TL, where *a* and *b* are parameters. The maximum likelihood ratio test was used to examine the difference in the BW–TL relationships among sexes.

## 3. Results

### 3.1. Global Distribution

**Figure 2:**
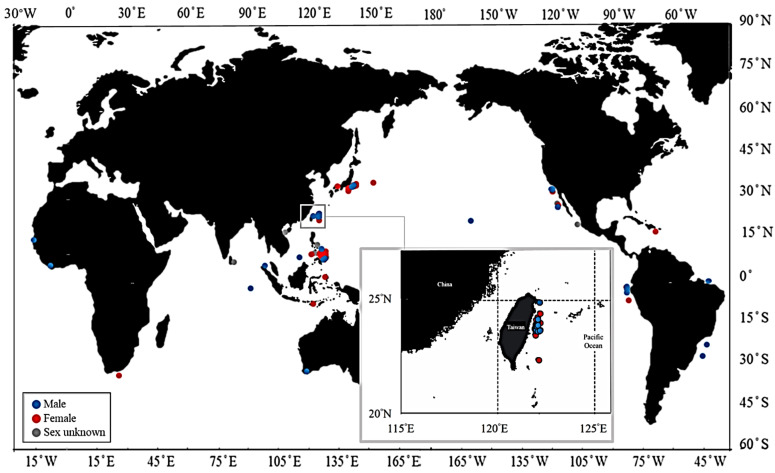
Records of *M. pelagios* in the world and in the eastern Taiwan waters.

**Figure 3:**
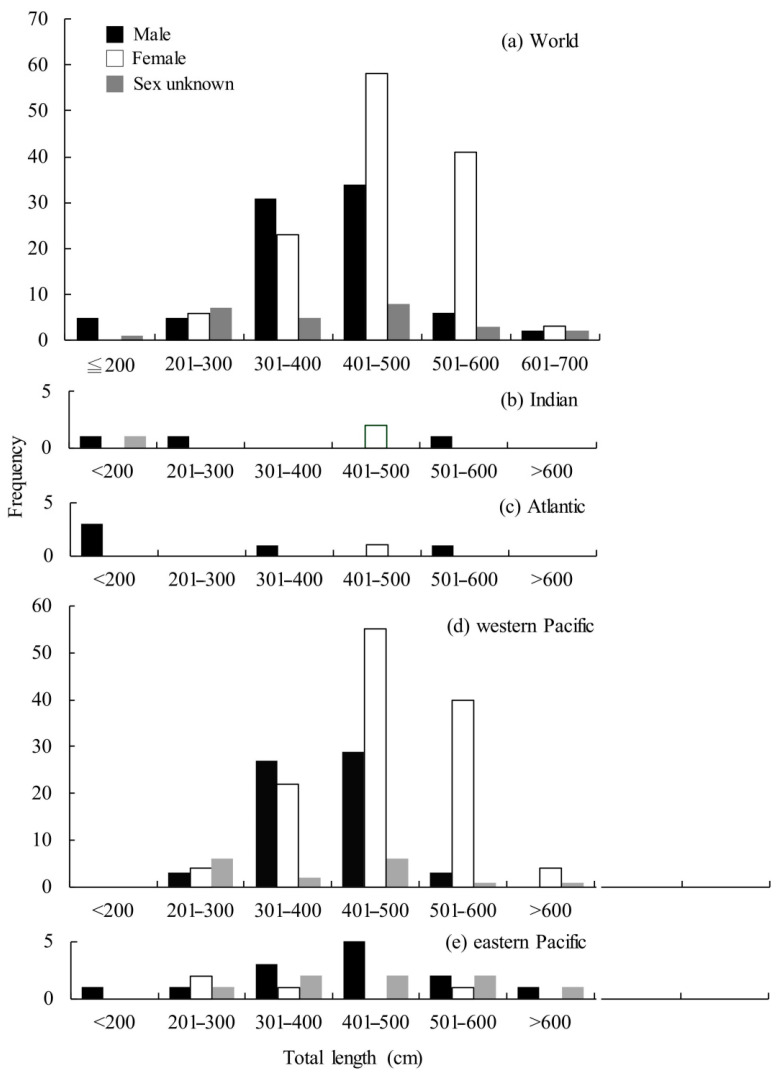
(**a**) Total length frequency of *M. pelagios* in the (**a**) three oceans, (**b**) Indian Ocean, (**c**) Atlantic Ocean, (**d**) Western Pacific Ocean, and (**e**) Eastern Pacific Ocean.

A total of 261 *M. pelagios* individuals recorded from 15 November 1976 to 7 August 2020 were analyzed in this study (Table S1). The majority of sharks were recorded from the western Pacific (*n* = 214), followed by the eastern Pacific (*n* = 35), with only six specimens being recorded each from the Atlantic and Indian oceans (Figure 2). Females represented slightly more than half (51%) of all sharks recorded with a breakdown by sex, if known, revealing a total of 132 females (226–710 cm TL) and 87 males (176.7–690.2 cm TL), with the sex unknown for 42 individuals (180–530 cm TL) (Figure 3a). The TL for all females was mostly between 400 and 500 cm, followed by 501 and 600 cm and 301 and 400 cm (Figure 3a). The TL for all males was mostly between 401 and 500 cm, followed closely by 301 and 400 cm (Figure 3a). Females represented the majority of records for the western Pacific (female:male = 125:65) compared with males, but more males (female:male = 4:14) were reported in the eastern Pacific (Figure 3).

### 3.2. Size and Sex Distribution in the Three Oceans

The length frequency of *M. pelagios* was estimated by three oceans, different groups of size and sex found tended to occur in different waters. The small individuals (<200 cm TL) were found more often but middle–large size individuals (250–400 cm TL) were few in the Indian and Atlantic Oceans (Figure 3b,c). More large males (≥500 cm TL) appeared in the eastern Pacific Ocean, but more large females (≥500 cm TL) in the western Pacific Ocean (Figure 3d,e).

**Figure 4:**
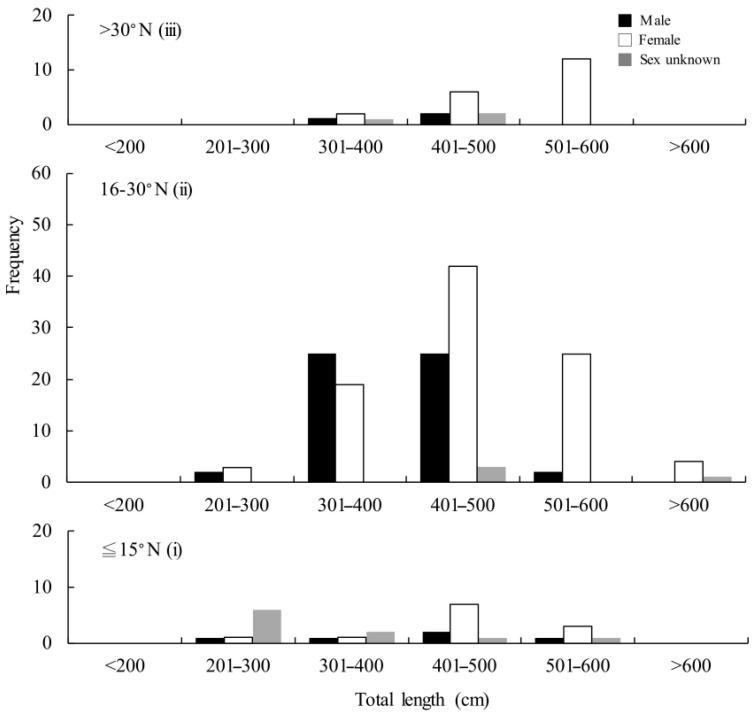
The length frequency of *M. pelagios* in the western Pacific Ocean.

Furthermore, because there was no record from the Southwestern Pacific, the records from the western North Pacific Ocean were divided by latitude, (i) ≤15° N, (ii) 15–30° N, and (iii) >30° N, and the ratios of females from zones (i) to (iii) were 43% (*n* = 28), 58% (*n* = 158), and 75% (*n* = 28), respectively (Figure 4). In zone (i), the length of *M. pelagios* ranged from ~300 to 549 cm TL for males (*n* = 5), 226 to 550 cm TL for females (*n* = 12), and 213 to 480 cm TL for sex unknown (*n* =11); the mean TL was 418 ± 114 cm TL (Figure 4). In zone (ii), the length ranged from 250 to 570 cm TL for males (*n* = 57), 247 to 710 cm TL for females (*n* = 92), and 470 to 700 cm TL for unknown sex (*n* = 9); the mean TL was 446 ± 80 cm TL (Figure 4). In zone (iii), the length ranged from 400 to 425 cm TL for males (*n* = 3), 346.6 to 577 cm TL for females (*n* = 21), and 247 to 710 cm TL for sex unknown (*n* = 4); the mean TL was 496 ± 74 cm TL (Figure 4).

### 3.3. BW–TL Relation and Conversion Equations

**Figure 5:**
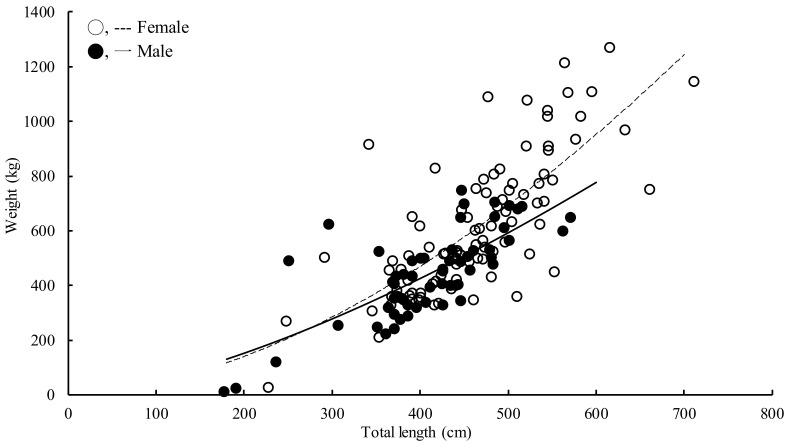
Total length–weight relationship of *M. pelagios*.

The maximum likelihood test indicated that there was a significant difference in the BW–TL between sexes (Chi-square = 7.92, critical value = 5.99, *p* < 0.05), and the sex-specific BW–TL relationships were estimated as follows (Figure 5):

BW = 0.014TL^1.74^ (females, *r*^2^ = 0.94, *n* = 93, *p* < 0.001) (1)

BW = 0.057TL^1.49^ (males, *r*^2^ = 0.95, *n* = 58, *p* < 0.001) (2)

The linear relationships among measurements were expressed as follows:

TL = 1.131PCL + 86.731 (*r*^2^ = 0.865, *n* = 126, *p* < 0.05) (3)

TL = 1.257FL + 1.407 (*r*^2^ = 0.975, *n* = 10, *p* < 0.05) (4)

TL = 4.236MW − 22.461 (*r*^2^ = 0.946, *n* = 3, *p* = 0.149) (5)

TL = 3.520D1A + 330.06 (*r*^2^ = 0.068, *n* = 61, *p* < 0.05) (6)

TL = 4.948D1B + 280.04 (*r*^2^ = 0.206, *n* = 54, *p* < 0.05) (7)

TL = 5.959P1A − 35.27 (*r*^2^ = 0.876, *n* = 9, *p* < 0.05) (8)

TL = 2.988CDM + 123.62 (*r*^2^ = 0.861, *n* = 6, *p* < 0.05) (9)

where BW is the body weight, TL is the total length, PCL is the precaudal length, FL is the fork length, MW is the mouth width, D1A is the 1st dorsal fin anterior margin, D1H is the 1st dorsal fin height, D1B is the 1st dorsal fin base, P1A is the pectoral fin anterior margin, and CDM is the caudal fin dorsal margin. For consistency, the lengths from other studies that were not reported in TL were converted to TL using above equations (Table S1).

### 3.4. Spatial–Temporal Distribution

#### 3.4.1. Horizontal Distribution

**Figure 6:**
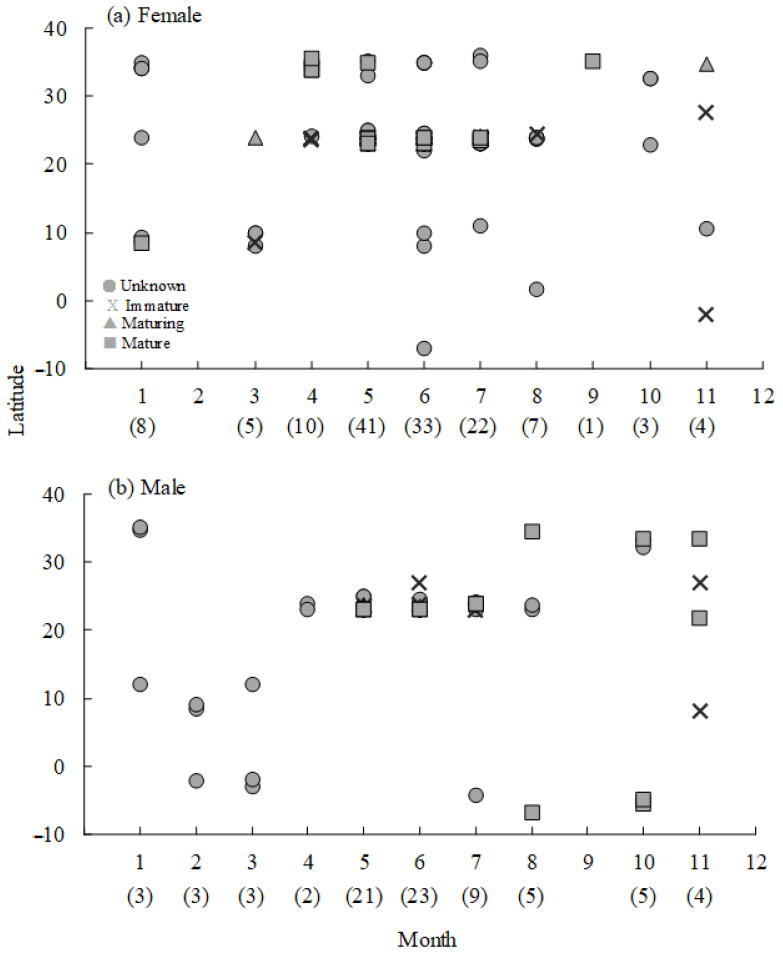
The monthly latitudinal occurrence in the Pacific Ocean of *M. pelagios* for (**a**) females and (**b**) males, number in the parentheses brackets was individual number.

According to the landing records of *M. pelagios* around the world, no immature individual landed at latitudes higher than 30°, while mature individuals were widely distributed (35° N–7° S). To further investigate the spatial–temporal distribution of *M. pelagios*, we eliminated data from the Indian and Atlantic Oceans and uncertain data from the Pacific Ocean. Figure 6 shows the monthly latitudinal occurrence of sex-specific *M. pelagios* at different maturity stages in the Pacific Ocean. Females appeared sporadically in the western Pacific Ocean from January to March and appeared in the higher latitude area (mainly in zone ii) from April to August (Figure 6a). In September, only one female was found in the high latitude area, and the distribution separated in the eastern and western Pacific Ocean after October (Figure 6a). On the other hand, male *M. pelagios* were found mainly in lower latitude waters in both the eastern and western Pacific Oceans from January to March. Males were mostly found in the middle latitude area from April to August (Figure 6b). There was no record for males in September, but mature males occurred in the eastern Pacific Ocean, and immature males occurred in the western Pacific Ocean in October and November (Figure 6b).

#### 3.4.2. Vertical Distribution

**Figure 7:**
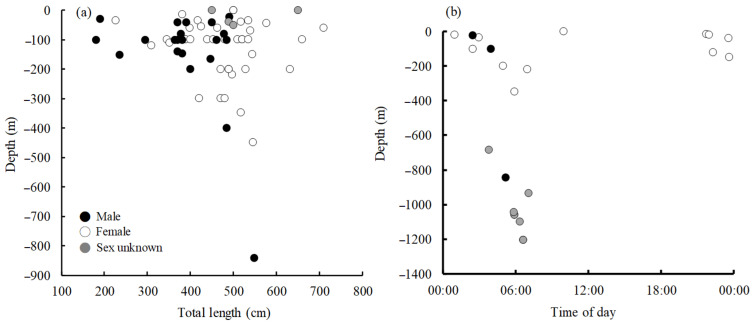
Vertical movement of *M. pelagios*. (**a**) The *M. pelagios* caught or sighted from different depths in size (*n* = 64). (**b**) Temporal vertical movement of *M. pelagios* (*n* = 23).

Figure 7a shows the 64 *M. pelagios* caught (*n* = 60) or sighted (*n* = 4) from different depths in size. Individuals < 300 cm TL were only found in the shallower water column (no deeper than 200 m), and large individuals were found at all water depths. In addition, the temporal vertical movements of 23 *M. pelagios* indicated that sharks tended to occur in deep water at dawn (00:00–06:00 a.m.) and then appeared in shallow water at dusk (18:00–00:00) (Figure 7b). However, one female was recorded around noon, which was a sighting event in which the individual was attacked by a whale.

## 4. Discussion

This study provided the first complete and detailed landing records of *M. pelagios* and its spatial–temporal distribution information. The results derived from this study can be used as a reference for future studies on the ecology, conservation, and management of this species.

In this study, we found that the body size of female *M. pelagios* was larger than that of males. One possible reason for this is that females need more space in the coelom to carry large and well-developed pups. Another reproductive strategy was also considered: females will be more reproductively fit through their growth, larger females may delivery more pups [31,32]. Although no pregnant *M. pelagios* has ever been found, the observation of its gonad structure showed that it was very similar to *C. maximus* [33]. Additionally, the smallest free-swimming *M. pelagios* was 176.7 cm TL. One convincing inference was that *M. pelagios* is an aplacental viviparous species, delivering a few well-developed pups, which is similar to *C. maximus* [11,34]. The ovary and uterus of female *M. pelagios* may become heavy when they reach the mature stage, leading to a length–weight relationship difference between sexes (Figure 4).

Parameter b of the length–weight equation of *M. pelagios* is far smaller than the value (2.5–3.0) commonly known from sharks [35]. According to the data for allometric equations having been weighed by scientists and fishermen associations, one possible reason for this is that *M. pelagios* is an engulfment filter feeder, the mechanism of energy use such as the metabolic rate and growth may differ from other shark species, leading to different allometric equation results compared with other species [14,36]. The linear regression analysis showed that TL had a good correlation with PCL, FL, P1A, and CDM, but the small sample size for FL, P1A, and CDM remains to be further enforced. On the other hand, the formulas of TL-MW, TL-D1A, and TL-D1B were for reference only due to either a limited sample size or low correlation. However, considering the rarity of *M. pelagios*, this information would still be useful. Furthermore, we attempted to estimate the “~9 m TL male” from Martínez-Ortiz et al. [5] by using the TL-CDM formula; pursuant to “1700 mm measured at the dorsal margin of the caudal fin”, this male had a 631.58 cm TL rather than ~9 m TL. This result indicated that the regression formulas from the present study provided an opportunity to validate uncertain data regarding *M. pelagios*. Further regression data should be collected from more individuals to be more convincing.

In this study, no evidence indicated that the population of *M. pelagios* is female biased due to 16% (*n* = 42) sexually unknown records. Some studies have indicated that the sexual segregation of elasmobranchs usually leads to sexual bias when under local investigation [37,38]. In this study, we integrated diverse record resources, including academic journals, conference reports, and public online resources, and the female ratio was 51%, close to half of the records. Additionally, *M. pelagios* is considered a panmictic population with no genetic structure, showing the ability to move across oceans [23]. The length frequency showed that small (≤200 cm TL) free-swimming *M. pelagios* (*n* = 4) were found only in the Indian and Atlantic Oceans, indicating a potential nursery area in these waters (Figure 3b,c). On the other hand, the males (*n* = 14) were more than three times as abundant as females (*n* = 4) in the eastern Pacific Ocean, but females were notably more abundant in the western North Pacific. Both the mean body size and the ratio of large females increased when the latitude was higher. Recent studies have shown that large shark species usually have sexual segregation behavior because (1) females escape forced mating by mature males, (2) to avoid consuming the same prey resources, and (3) gravid females move to habitats that offer stable resources, through which they can gain more energy for offspring from predation [39,40]. A significant sexual bias of reef manta rays (*Mobula alfredi*) was found in southern Mozambique, and female *M. alfredi* uses this habitat as the breeding and birthing grounds [41]. During the mating season, male shortfin mako sharks (*Isurus oxyrinchus*) harass females that lead to fitness consequences, which reflect avoidance behavior [40]. White sharks (*Carcharodon carcharias*) near the Neptune Islands experience segregation due to different physiological strategies, females are absent in the breeding season and only return in the feeding period to increase the growth rate of pups [37]. Large shark species usually have large spatial-scale segregation behavior, and our results showed that female *M. pelagios* mainly inhabit the western Pacific Ocean, while males prefer to inhabit the eastern Pacific Ocean. However, the mechanisms resulting in sex segregation need further investigation in the future.

Immature individuals were found only between 30° N and 30° S, but mature *M. pelagios* could not only move toward higher latitude waters, but also have the ability to dive deeper, where the water temperature is lower (Figure 6 and Figure 7). Previous studies have suggested that many Lamnidae sharks, such as *I. oxyrinchus*, big-eye thresher (*Alopias superciliosus*), and pelagic thresher (*A. pelagicus*), have some capability of endothermic regulation; they could conserve heat and arrange their body temperature well to protect against low temperatures (high latitude or winter) environments [42,43]. The most famous endothermic species are the porbeagle (*Lamna nasus*) and salmon shark (*L. ditropis*), which are usually distributed at latitudes higher than 40°; however, limited by thermal inertia, young individuals can only remain in moderate temperature areas until they mature [44,45]. The distribution pattern of *M. pelagios* based on latitude or water depth in this study showed that mature individuals have the ability to protect themselves against low-temperature water compared with immature individuals, although there is a lack of evidence to verify whether *M. pelagios* is an endothermic species.

Records from the western North Pacific Ocean were mostly bycatch from fisheries (85%). According to fishermen, the occurrence of *M. pelagios* seems to be seasonal, some large-mesh drift net fishing vessels in Taiwan operate year-round but do not catch any *M. pelagios* from September to March. On the other hand, M. pelagios was mainly recorded between October and March in the eastern Pacific Ocean (74%). These results may indicate that *M. pelagios* move between eastern and western Pacific waters. Large sharks are able to move across the Ocean, a tracking study provided evidence of the trans-Pacific migration of the whale shark (*Rhincodon typus*). One female *R. typus* individual was tracked from the eastern (Panama) to the western (Mariana Trench) Pacific Ocean [46]. In addition, the monthly latitudinal occurrence of *M. pelagios* was slightly different between females and males. In the spring, both sexes from lower latitudes (zone i) moved toward middle latitude water (zone ii), but females also went further to the high latitude area (zone iii) in the western North Pacific Ocean. In the summer, *M. pelagios* was dispersed but mainly found in zone ii around Taiwanese waters in the western North Pacific Ocean. Afterward, *M. pelagios* was absent in early autumn (September); by October, females appeared in zone iii and the eastern Pacific, and males mainly appeared in the eastern Pacific. The records from winter were few, but included *M. pelagios* from both the eastern and western North Pacific Oceans and different latitudes.

Geographically, the movement of *M. pelagios* seems to be related to the current flow in the North Pacific Ocean. Many studies have suggested that the migration or movement of large marine animals relates to the current, and they benefit from the current, such as for moving, spawning, or foraging [47,48]. There are different names of current in the North Pacific Gyre (NPG) according to their position and characteristics, including the Kuroshio Current (KC), which is warm, less productive flows pass through the western Pacific Ocean, and extends from the Philippines to Taiwan and Japan year-round. The intensity of the KC increases from May to August; during this period, many migrating fish species are transported by KC, passing through eastern Taiwanese waters toward northern Japan. The KC merges with the Oyashio Current (OC) at approximately 35° N, forming a good feeding and fishing ground and, finally, turning toward the east across the North Pacific Ocean [49]. There was evidence shown that large sharks such as *R. typus* prefer to give birth or aggregate in warm waters [50,51]. *Megachasma pelagios* migrate northward with the KC in the late spring to summer offshore of Taiwan. Tang et al. [52] suggested that the subsurface Kuroshio water on the shelf along the east coast of Taiwan indicated upwelling and nutrient transport, which could explain why the *M. pelagios* we observed from Taiwan were mostly full with prey in their stomach. Therefore, the middle western Pacific Ocean may be the feeding grounds of *M. pelagios* in the spring and summer; moving northward to 35° N, some females remain and other individuals change direction toward the eastern Pacific Ocean in early autumn and arrive after October. When arriving in the eastern Pacific Ocean, *M. pelagios* move southward with the California Current (CC) until they meet the Peru Current (PC) and then turn to cross the Pacific again toward the western side, which returns to Indonesian or Philippine waters. This inference was based on the lack of genetic structure and panmictic populations in *M. pelagios*, but further study in addition to the genetic study of Liu et al. [23], such as tagging or analyzing more specimens from different oceans, should be conducted to verify this hypothesis.

Vertical migration behavior has been verified for *M. pelagios* since 1997. One 490 cm TL male was attached with acoustic transmitters and tracked for 50.5 h in the eastern Pacific (southern California), and the results indicated that *M. pelagios* has a very specific vertical movement during dawn and dusk [19]. Sharks make vertical movements for different purposes, e.g., *R. typus* spends time daily at the surface to gain energy for thermoregulation [53]; the basking shark (*Cetorhinus maximus*) spends half a day in deep water (800–1000 m) and reduces depths gradually, indicating foraging behavior [54]. However, many studies have shown that even the same species may have different horizontal or vertical movement patterns [48,53,55]. As only one *M. pelagios* individual was successfully tracked in the past, we integrated historical time–depth records of *M. pelagios*. In the present study, the daily vertical movement of *M. pelagios* was found in multiple individuals. The shallow–deep water movement was extremely significant from dusk to dawn, but one record was found at approximately 10 am (Figure 7b). Amorim et al. [56] noted that one *M. pelagios* was sighted with three sperm whales (*Physeter macrocephalus*), and there was some scarring on the fin and gills of the shark, indicating that it may have been attacked or traced by the sperm whales; therefore, it came to the surface. In addition to the sighting of this individual, other studies have shown a similar temporal vertical movement pattern of *M. pelagios* with acoustic techniques [19]. However, most data were operating depth recorded by the Taiwan large-mesh drift net fishery and NOAA Fisheries; the actual catching depth remains to be further elucidated. According to previous studies, as a filter-feeding shark species, *M. pelagios* seems to prefer euphausiid shrimp. Sawamoto and Matsumoto [15] observed the stomach composition of one female *M. pelagios*, which was caught by a seine net near Japan, and euphausiids (*Euphausia pacifica*) were the main prey of *M. pelagios* [20]. Nakagawa et al. [57] found that *E. pacifica* migrate to the surface at night (20:30), move down to approximately 100 m at midnight (00:30), and move toward deeper water (150–300 m) after dawn (06:00). The vertical movement patterns of *M. pelagios* and *E. pacifica* seem very similar, indicating that the vertical movement of *M. pelagios* may be related to its foraging behavior.

The notable number of *M. pelagios* landing records from Taiwanese waters compared with those elsewhere may be attributed to the cooperation between fishermen, research institutes, and the Fishery Agency (Figure 2). Due to the catch and report system, we were able to measure *M. pelagios* at the market. The large-mesh drift net fishery usually operates on the east coast of Taiwan from April to August and targets *M. mola* and *M. lanceolatus* at night. The fishermen change different fishing gear in other months in order to catch other species, e.g., striped bonito (*Sarda orientalis*). Previous studies have suggested that oceanic sunfish movement vertically depends on the temperature and depth of the mixed layer. Moreover, oceanic sunfish also move to shallower water during the night and back to deeper water at dawn, which is similar to *M. pelagios* [58,59]. Therefore, *M. pelagios* may be accidentally caught by the drift net due to sharing the same vertical movement as molas. Additionally, the catch and report system plays an important role; nearly 40% of the records from the Philippines and Indonesia are either stranded or sexually unknown because of the scattered islands, which prevents the information from being transmitted effectively. To better understand the information of *M. pelagios*, the reporting system or open platform should be designed and propagated, especially for waters with potential nursery grounds.

The data collected on the spatial–temporal movement of *M. pelagios* provide important insights into their vertical and geographic migration behaviors. This study was the first to include different body part measurements of multiple *M. pelagios* individuals using the same standards. Additionally, we integrated the results from previous studies, refined the data records presented in supplementary materials (Table S1), and established conversion equations for future research. Furthermore, we updated the catch records from Taiwanese waters, including the 250–400 cm TL individuals, integrated the missing records from April to October, and included the vertical movement data in this study.

## 5. Conclusions

**Figure 8:**
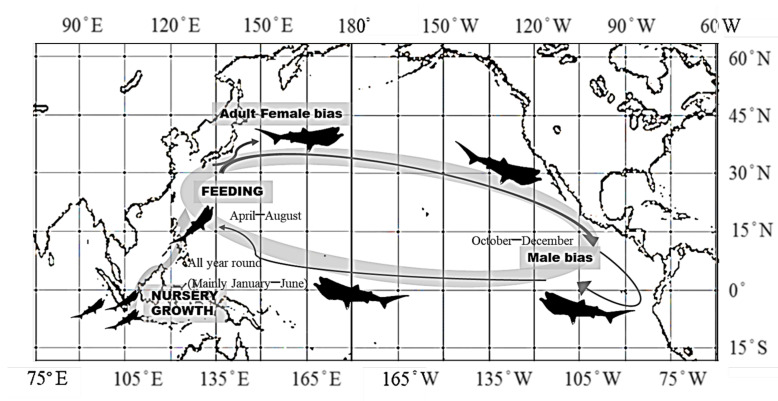
Schematic *M. pelagios* spatial–temporal distribution model in the Pacific Ocean proposed by this study.

One hypothesis was proposed in this study: *M. pelagios* give birth in the eastern Indian Ocean near the Philippines and Indonesia; during growth, they move northward to the western Pacific Ocean, joining the NPG in the spring and arriving in Taiwanese waters, foraging on planktonic prey from late spring to summer (Figure 8). Some mature females, which can withstand cool temperatures, keep following the KC and arrive in Japanese waters in the spring. In late summer, females remain in the water around Japan, and males remain across the Pacific Ocean toward the eastern side by following the North Pacific Current (NPC), indicating sexual segregation. *M. pelagios* arrive in Californian waters in late summer or autumn by following the CC and PC south to Mexico, Ecuador, and Peru. Afterward, some *M. pelagios* may follow the North Equatorial Current (NEC) across the Pacific again toward the western side, thereby returning to Indonesian or Philippine waters. However, (1) how does the Atlantic Ocean serve as a potential nursery area for *M. pelagios*? (2) Where do males and females mate? (3) Where do the gravid females go? These questions remain poorly known and need further study. The catch and retention of *M. pelagios* have been banned in Taiwan, fishermen have to release the shark no matter if it is alive or dead [30]. Therefore, data collection and biological study, such as reproduction and age growth, will be difficult in the future. Future studies, such as satellite tracking or international data exchange, would help confirm our hypothesis.

## Supplementary Materials

The following are available online at https://www.mdpi.com/article/10.3390/ani11102947/s1, Table S1: complete megamouth shark records from November 1976 to August 2020 [4,60,61,62,63,64,65,66,67,68,69,70,71,72,73,74,75,76,77,78,79].
