# Supplementary material for: Spatial–Temporal Distribution of Megamouth Shark, Megachasma pelagios, Inferred from over 250 Individuals Recorded in the Three Oceans
Source: Animals (Basel). 2021 Oct 12;11(10):2947. doi: 10.3390/ani11102947 (PMC8532755; doi:10.3390/ani11102947)
Supplement: Supplementary file 1 [file animals-11-02947-s001.zip › animals-1390701-supplementary.pdf]

animals

Supplementary Materials

Spatial–Temporal Distribution of Megamouth Shark, *Megachasma pelagios*, Inferred from over 250 Individuals Recorded in the Three Oceans

Chi-Ju Yu [1,2], Shoou-Jeng Joung [1,3,*], Hua-Hsun Hsu [4], Chia-Yen Lin [1], Tzu-Chi Hsieh [1], Kwang-Ming Liu [3,5,6] and Atsuko Yamaguchi [2]

1. Department of Environmental Biology and Fisheries Science, National Taiwan Ocean University, Keelung, 20224, Taiwan; wing13260@gmail.com (C.-J.Y.); skinomotohiko@gmail.com (C.-Y.L.); andy760327@gmail.com (T.-C.H.)
2. Graduate School of Fisheries Science and Environmental Studies, Nagasaki University, Nagasaki, 852-8521, Japan; y-atsuko@nagasaki-u.ac.jp
3. George Chen Shark Research Center, National Taiwan Ocean University, Keelung, 20224, Taiwan; kmliu@mail.ntou.edu.tw
4. Marine Studies Section, Center for Environment and Water, Research Institute, King Fahd University of Petroleum and Minerals, Dhahran, 31261, Saudi Arabia; hsuhuahsun@yahoo.com.tw
5. Institute of Marine Affairs and Resource Management, National Taiwan Ocean University, Keelung 20224, Taiwan
6. Center of Excellence for the Oceans, National Taiwan Ocean University, Keelung, 20224, Taiwan

* Correspondence: f0010@mail.ntou.edu.tw

Table S1. A complete megamouth shark records from November 1976 to August 2020.

No	year	month	Country	Sex	TL (cm)	Weight (kg)	Method	Reference/ Sources
1	1976	11	USA	M	446	750	Entangling	[1]
2	1984	11	USA	M	449	700	Gill net	[2]
3	1988	8	Australia	M	515	690	Stranded	[3]
4	1989	1	Japan	M	>400	-	Stranded	[4]
5	1989	6	Japan	-	~490	-	Set net	[5]
6	1990	10	USA	M	490	-	Gill net	[6]
7	1994	11	Japan	F	471	790	Stranded	[7]
8	1995	5	Senegal	M	180	-	Purse seine	[8]
9	1995	9	Brazil	M	190	24	Longline	[9]
10	1997	4	Japan	F	544	1040	Purse seine	[10]
11	1998	2	Philippines	M	~549	-	Gill net	[9]
12	1998	4	Japan	F	520-549	-	Set net	[9]
13	1998	8	Indonesia	F	~500	-	Sighting	[9]
14	1999	10	USA	F	518	-	Gill net	[3]
15	2001	10	USA	M	690.24*	-	Gill net	[3]
16	2002	1	East Indian Ocean	M	235	120	Purse seine	[3]
17	2002	4	South Africa	F	~460	260	Stranded	[12]
18	2003	1	Philippines	F	497	-	Gill net	[3]
19	2003	5	USA	-	610-760	-	Sighting	[3]
20	2003	7	Taiwan	M	250	490	-	[13]
21	2003	8	Japan	M	425	460	Purse seine	[14]
22	2004	3	Ecuador	M	529.35*	600	Gill net	[3]
23	2004	3	Indonesia	M	176.7	14	Stranded	[15]
24	2004	4	Japan	F	563	1215	Stranded	[16]
25	2004	4	Japan	F	~490	-	Set net	[16]
26	2004	11	Philippines	F	504	~1000	Stranded	[3]

27	2005	1	Japan	F	528	-	Purse seine	[3]
28	2005	1	Philippines	F	417	~1000	Net	[3]
29	2005	4	Taiwan	-	-	580-630	Trawl net	[13]
30	2005	5	Taiwan	-	-	580-680	Trawl net	[13]
31	2005	5	Taiwan	F	487	689	Trawl net	[13]
32	2005	5	Taiwan	F	483	807	Trawl net	[13]
33	2005	5	Taiwan	-	350-400	400-500	Drift net	[28]
34	2006	1	Philippines	F	500	750	Gill net	[3]
35	2006	3	Philippines	F	226	60-80	Gill net	[3]
36	2006	3	off China Sea	-	470	650	-	[28]
37	2006	5	Japan	F	567	1105	Set net	[3]
38	2006	11	Mexico	F	227	27	Gill net	[18]
39	2007	5	Philippines	-	249-250	40-50	-	[19]
40	2007	6	Japan	F	540	-	Set net	[3]
41	2007	7	Japan	F	367	361	Purse seine	[20]
42	2007	9	Mexico	-	-	-	Gill net	[18]
43	2007	9	Philippines	-	274	-	Stranded	[19]
44	2008	6	Taiwan	M	-	>200	-	[13]
45	2008	7	Taiwan	M	500-550	870	-	[13]
46	2008	9	Philippines	-	213	-	Stranded	[19]
47	2009	3	Philippines	M	400	500	Gill net	[19]
48	2009	6	Taiwan	F	390	350	-	[13]
49	2009	7	Brazil	M	539	-	Stranded	[21]
50	2009	11	Mexico	M	180-200	-	Net	[18]
51	2010	4	off China Sea	M	~400	>1000	-	[28]
52	2010	6	Taiwan	-	700	~770	-	[28]
53	2010	6	Japan	F	570	-	-	[22]
54	2010	-	Philippines	-	-	-	-	[19]
55	2011	1	Japan	F	~500	-	Set net	[23]

56	2011	5	Japan	-	>300	-	set net	[24]
57	2011	6	Mexico	M	300	-	Gill net	[25]
58	2011	6	Japan	F	540	-	Set net	[26]
59	2011	7	Japan	F	346.6	-	Set net	[22]
60	2011	11	Ecuador	F	243	-	Gill net	[27]
61	2012	1	off China Sea	F	565-570	1150-1250	-	[28]
62	2012	3	Ecuador	M	-	-	Gill net	[27]
63	2012	7	Sri Lanka	-	180	-	Gill net	[28]
64	2012	10	Taiwan	F	571.93*	800-900	-	[29]
65	2012	11	USA	-	-	-	Gill net	NOAA Fisheries (personal communication)
66	2012	11	USA	-	-	-	Gill net	NOAA Fisheries (personal communication)
67	2013	1	Japan	-	-	-	Set net	[30]
68	2013	1	Japan	F	450	-	Set net	[30]
69	2013	1	Japan	F	-	-	-	[19]
70	2013	4	Taiwan	F	387	365	Drift net	This study
71	2013	4	Taiwan	F	250	200-300	Drift net	This study
72	2013	4	Taiwan	F	373	383	Drift net	This study
73	2013	5	Taiwan	M	500	565	Drift net	This study
74	2013	5	Taiwan	F	476	1090	Drift net	This study
75	2013	5	Taiwan	M	368	413	Drift net	This study
76	2013	5	Taiwan	M	385	328	Drift net	This study
77	2013	5	Taiwan	F	413	408	Drift net	This study
78	2013	5	Taiwan	F	400	356	Drift net	This study
79	2013	5	Taiwan	F	545	910	Drift net	This study
80	2013	5	Taiwan	F	524	516	Drift net	This study
81	2013	5	Taiwan	F	552	452	Drift net	This study
82	2013	5	Taiwan	F	509	360	Drift net	This study
83	2013	5	Taiwan	M	395	320	Drift net	This study
84	2013	5	Taiwan	M	363	320	Drift net	This study

85	2013	5	Taiwan	F	453	650	Drift net	This study
86	2013	5	Taiwan	F	426	516	Drift net	This study
87	2013	6	Taiwan	M	380	348	Drift net	This study
88	2013	7	Taiwan	F	463	549	Drift net	This study
89	2013	7	Taiwan	F	398	348	Drift net	This study
90	2013	7	Taiwan	M	484	653	Drift net	This study
91	2013	7	Taiwan	F	710	1147	Drift net	This study
92	2013	9	Japan	F	577	-	-	[31]
93	2013	11	USA	-	-	-	Gill net	NOAA Fisheries (personal communication)
94	2013	11	USA	-	-	-	Gill net	NOAA Fisheries (personal communication)
95	2014	2	Ecuador	M	582-650	-	Gill net	[27]
96	2014	3	Philippines	F	>400	-	Stranded	[19]
97	2014	4	Japan	F	446	677	Set net	[30]
98	2014	5	Taiwan	F	341	916	Drift net	This study
99	2014	5	Taiwan	F	352	210	Drift net	This study
100	2014	5	Taiwan	F	660	752	Drift net	This study
101	2014	5	Taiwan	M	478	532	Drift net	This study
102	2014	5	Taiwan	M	377	277	Drift net	This study
103	2014	6	Taiwan	F	517	734	Drift net	This study
104	2014	6	Taiwan	M	370	355	Drift net	This study
105	2014	6	Taiwan	M	390	490	Drift net	This study
106	2014	6	Taiwan	M	370	296	Drift net	This study
107	2014	6	Philippines	-	>300	~1000	Net	[19]
108	2014	6	Philippines	F	548	400-500	-	[19]
109	2014	8	Taiwan	F	366	330	Drift net	This study
110	2014	12	Japan	-	~500	-	Set net	[32]
111	2015	1	Philippines	M	457	-	Net	[19]
112	2015	3	Philippines	F	~365	-	Net	[19]
113	2015	5	Taiwan	F	-	-	Drift net	This study

114	2015	5	Taiwan	F	345	307	Drift net	This study
115	2015	5	Taiwan	M	-	540	Drift net	This study
116	2015	6	Vietnam	-	~500	540	Stranded	[19]
117	2015	6	Taiwan	F	386	510	Drift net	This study
118	2015	6	Taiwan	M	456	456	Drift net	This study
119	2015	6	Taiwan	F	535	625	Drift net	This study
120	2015	6	Taiwan	F	521	1077	Drift net	This study
121	2015	6	Taiwan	F	440	523	Drift net	This study
122	2015	7	Taiwan	F	247	271	Drift net	This study
113**	2015	7	Taiwan	F	290	502	Drift net	This study
123	2015	11	Philippines	-	>300	-	Net	[19]
124	2015	12	Mexico	-	530	-	Stranded	[19]
125	2016	4	Japan	F	~500	-	Set net	[33]
126	2016	5	Taiwan	-	-	-	Drift net	This study
127	2016	5	Taiwan	-	-	-	Drift net	This study
128	2016	11	Peru	-	~350	-	Gill net	[34]
129	2016	12	Puerto Rico	F	457	-	Stranded	[35]
130	2017	5	Philippines	-	~300	~600	Stranded	[19]
131	2017	5	Taiwan	F	440	498	Drift net	This study
132	2017	5	Taiwan	M	484	705	Drift net	This study
133	2017	5	Taiwan	F	480	617	Drift net	This study
134	2017	5	Japan	F	540	-	Set net	[36]
135	2017	5	Japan	F	510	-	Set net	[19]
136	2017	5	Taiwan	M	295	625	Drift net	This study
137	2017	5	Taiwan	M	460	528	Drift net	This study
138	2017	6	Philippines	F	400-500	-	Stranded	[19]
139	2017	6	Taiwan	F	520	910	Drift net	This study
140	2017	6	Taiwan	F	390	653	Drift net	This study

141	2017	6	Taiwan	M	370	407	Drift net	This study
142	2017	7	Taiwan	F	490	827	Drift net	This study
143	2017	7	Indonesia	F	~500	-	Sighting	[19]
144	2017	7	Taiwan	F	632	969	Drift net	This study
145	2017	8	Taiwan	F	470	567	Drift net	This study
146	2017	8	Taiwan	F	480	432	Drift net	This study
147	2017	8	Taiwan	F	420	335	Drift net	This study
148	2017	8	Taiwan	F	470	498	Drift net	This study
149	2018	2	Philippines	M	434	-	Gill net	[19]
150	2018	3	Philippines	F	>400	-	Net	[19]
151	2018	4	Taiwan	F	534	773	Drift net	This study
152	2018	5	Taiwan	-	495	613	Drift net	This study
153	2018	5	Taiwan	M	306	254	Drift net	This study
154	2018	5	Taiwan	F	437	~1200	Drift net	Paul J Clerkin (personal communication)
155	2018	5	Taiwan	F	440	500	Drift net	This study
156	2018	5	Taiwan	F	385	420	Drift net	This study
157	2018	5	Taiwan	F	415	329	Drift net	This study
158	2018	5	Taiwan	M	433	490	Drift net	This study
159	2018	5	Taiwan	M	410	~650	Drift net	Paul J Clerkin (personal communication)
160	2018	5	Taiwan	F	565	~1200	Drift net	Paul J Clerkin (personal communication)
161	2018	5	Taiwan	F	428	520	Drift net	This study
162	2018	5	Taiwan	F	455	487	Drift net	This study
163	2018	5	Taiwan	F	364	456	Drift net	This study
164	2018	5	Taiwan	F	368	492	Drift net	This study
165	2018	5	Taiwan	M	500	692	Drift net	This study
166	2018	5	Taiwan	M	372	436	Drift net	This study
167	2018	5	Taiwan	F	400	373	Drift net	This study
168	2018	5	Taiwan	F	445	513	Drift net	This study
169	2018	5	Taiwan	F	390	372	Drift net	This study

170	2018	5	Taiwan	F	416	830	Drift net	This study
171	2018	5	Taiwan	M	570	649	Drift net	This study
172	2018	5	Taiwan	F	465	500	Drift net	This study
173	2018	5	Taiwan	F	472	540	Drift net	This study
174	2018	5	Taiwan	M	404	500	Drift net	This study
175	2018	5	Taiwan	M	436	530	Drift net	This study
176	2018	5	Taiwan	F	440	424	Drift net	This study
177	2018	5	Taiwan	M	360	223	Drift net	This study
178	2018	6	Taiwan	F	440	480	Drift net	This study
179	2018	6	Taiwan	F	463	755	Drift net	This study
180	2018	6	Taiwan	M	445	346	Drift net	This study
181	2018	6	Taiwan	F	417	417	Drift net	This study
182	2018	6	Taiwan	F	399	618	Drift net	This study
183	2018	6	Taiwan	F	493	716	Drift net	This study
184	2018	6	Taiwan	M	445	650	Drift net	This study
185	2018	6	Taiwan	M	380	440	Drift net	This study
186	2018	6	Taiwan	M	370	243	Drift net	This study
187	2018	6	Taiwan	M	453	508	Drift net	This study
188	2018	6	Taiwan	M	420	~300	Drift net	This study
189	2018	6	Taiwan	M	-	~220	Drift net	This study
190	2018	6	Taiwan	M	410	396	Drift net	This study
191	2018	6	Taiwan	M	406	338	Drift net	This study
192	2018	6	Taiwan	F	435	390	Drift net	This study
193	2018	7	Philippines	F	550	-	Stranded	[19]
194	2018	7	Taiwan	F	378	460	Drift net	This study
195	2018	7	Taiwan	F	544	1020	Drift net	This study
196	2018	7	Peru	M	364-411	-	Gill net	[34]
197	2018	7	Taiwan	F	460	348	Drift net	This study
198	2018	7	Taiwan	F	441	527	Drift net	This study

199	2018	7	Taiwan	M	425	330	Drift net	This study
200	2018	7	Taiwan	F	425	454	Drift net	This study
201	2018	7	Peru	-	370	-	Stranded	[34]
202	2018	8	Taiwan	M	434	400	Drift net	This study
203	2018	9	Brazil	M	>100	-	Stranded	[19]
204	2018	10	USA	-	-	-	Gill net	NOAA Fisheries (personal communication)
205	2018	10	USA	-	-	-	Gill net	NOAA Fisheries (personal communication)
206	2018	10	USA	-	-	-	Gill net	NOAA Fisheries (personal communication)
207	2018	10	Philippines	-	>200	-	Stranded	[19]
208	2018	10	Peru	-	>500	-	Gill net	[37]
209	2018	11	Philippines	M	~300	-	Stranded	[19]
210	2018	12	USA	-	-	-	Gill net	NOAA Fisheries (personal communication)
211	2019	1	Japan	M	~400	-	Stranded	[19]
212	2019	3	Peru	-	470	-	Gill net	[37]
213	2019	4	Philippines	-	~480	-	Stranded	[19]
214	2019	4	Taiwan	M	300-350	420	Drift net	This study
215	2019	5	Taiwan	M	495	612	Drift net	This study
216	2019	5	Taiwan	M	390	435	Drift net	This study
217	2019	5	Taiwan	F	503	633	Drift net	This study
218	2019	5	Taiwan	F	550	787	Drift net	This study
219	2019	5	Taiwan	F	496	558	Drift net	This study
220	2019	6	Taiwan	F	540	808	Drift net	This study
221	2019	6	Taiwan	M	385	289	Drift net	This study
222	2019	6	Taiwan	M	480	503	Drift net	This study
223	2019	6	Taiwan	M	442	403	Drift net	This study
224	2019	6	Taiwan	F	>500	998	Drift net	This study
225	2019	6	Peru	F	>300	-	Seine net	[34]
226	2019	6	Taiwan	M	445	487	Drift net	This study
227	2019	6	Taiwan	F	467	610	Drift net	This study

228	2019	6	Taiwan	F	474	740	Drift net	This study
229	2019	6	Taiwan	M	374	359	Drift net	This study
230	2019	6	Taiwan	F	497	671	Drift net	This study
231	2019	6	Peru	-	215	-	Gill net	[37]
232	2019	7	Philippines	-	~300	-	Net	[19]
233	2019	7	Taiwan	F	540	708	Drift net	This study
234	2019	7	Taiwan	M	424	408	Drift net	This study
235	2019	7	Taiwan	F	615	1270	Drift net	This study
236	2019	7	Taiwan	M	390	436	Drift net	This study
237	2019	7	Taiwan	M	352	525	Drift net	This study
238	2019	7	Taiwan	F	532	703	Drift net	This study
239	2019	7	Taiwan	F	439	401	Drift net	This study
240	2019	7	Taiwan	M	510	682	Drift net	This study
241	2019	7	Taiwan	M	350	248	Drift net	This study
242	2019	8	Taiwan	M	482	480	Drift net	This study
243	2019	8	Peru	M	375	-	Gill net	[37]
244	2019	8	Peru	M	400	-	Gill net	[37]
245	2019	10	USA	-	~450	-	Sighting	[19]
246	2019	10	Peru	M	>400	-	Gill net	[37]
247	2019	10	Peru	M	>400	-	Gill net	[37]
248	2020	3	Liberia	M	380	-	Gill net	[19]
249	2020	3	Taiwan	F	545	895	Drift net	This study
250	2020	5	Taiwan	F	423	439	Drift net	This study
251	2020	6	Philippines	-	~600	-	Stranded	[19]
252	2020	6	Japan	F	~600	-	Set net	[19]
253	2020	6	Taiwan	F	462	602	Drift net	This study
254	2020	6	Taiwan	F	582	1018	Drift net	This study
255	2020	6	Taiwan	M	378	354	Drift net	This study
256	2020	6	Taiwan	F	576	935	Drift net	This study

257	2020	6	Taiwan	F	594	1110	Drift net	This study
258	2020	6	Taiwan	F	482	526	Drift net	This study
259	2020	7	Taiwan	F	504	774	Drift net	This study
260	2020	7	Taiwan	F	409	542	Drift net	This study
261	2020	8	Taiwan	F	381	-	Longline	This study

References

1. Taylor, L.R.; Compagno, L.J.V.; Struhsaker, P.J. Megamouth—A new species, genus, and family of lamnoid shark (Megachasma pelagios, family Megachasmidae) from the Hawaiian Islands. *Proc. Calif. Acad. Sci.* **1983**, *43*, 87–110.
2. Lavenberg, R.J., and Seigel, J.A. The Pacific's megamystery—megamouth. *Terra*. **1985**, *23(4)*, 29–31.
3. Berra, T.M., and Hutchins, J.B.A specimen of megamouth shark, *Megachasma pelagios* (*Megachasmidae*) from Western Australia. *Rec. West. Aust. Mus*. **1990**, *14(4),* 651–656.
4. Nakaya, K. Discovery of a megamouth shark from Japan. Japan. *J. Ichthyol*. **1989**, *36*, 144–146.
5. Miya, M., Hirosawa, M., and Mochizuki, K. Occurrence of a megachasmid shark in Suruga Bay: photographic evidence. *J. Nat. Hist. Mus. Inst*., Chiba, **1992**, *2*: 41–44.
6. Nelson, D.R.; McKibben, J.N.; Strong, W.R.; Lowe, C.G.; Sisneros, J.; Schroeder, D.M.; Lavenberg, R.J. An acoustic tracking of a megamouth shark, Megachasma pelagios: A crepuscular vertical migrator. *Environ. Boil. Fishes* **1997**, *49*, 389–399, https://doi.org/10.1023/a:1007369619576.
7. Takada, K., Hiruda, H., Wakisaka, S., Mori, T., and Nakaya, K. Capture of the first female megamouth shark, *Megachasma pelagios*, from Hakata Bay, Fukuoka, Japan. In *Biology of the Magamouth Shark*. Yano, K., Morrissey, J. F., Yabumoto, Y., and Nakaya, K. Tokai University Press, Tokyo, Japan. 1997, 3–9.
8. Séret, B. Première capture d'un requin grande gueule (Chondrichthyes, Megachasmidae) dans l'Atlantique, au large du Sénégal. *Cybium* **1995**, *19*, 425–427.
9. Amorim, A.F.; Arfelli, C.A.; Castro, J.I. Description of a Juvenile Megamouth Shark, Megachasma pelagios, Caught off Brazil. *Environ. Boil. Fishes* **2000**, *59*, 117–123, https://doi.org/10.1023/a:1007609617773.
10. Yano, K., Yabumoto, Y., Tanaka, S., Tsukada, O., and Furuta, M. Capture of a mature female megamouth shark, *Megachasma pelagios*, from Mie, Japan. In *Proceedings of the 5th Indo-Pacific Conference*, *Soc. Fr. Ichtyol.*, Nouméa, New Caledonia. **1997**, 335–349.
11. Nakaya, K. Biology of the megamouth shark, Megachasma pelagios (Lamniformes: Megachasmidae). In Proceedings of the International Symposium, Into the Unknown, Researching Mysterious Deep-sea Animals, Okinawa, Japan, 2010; pp. 69–83.
12. Smale, M. J., Compagno, L. J. V., and Human, B. A. First megamouth shark from the western Indian Ocean and South Africa: news & views. *S. Afr. J. Sci.* **2002**, *98(7)*, 349–350.
13. Lee, P.F., and Shao, K.T. Two new records of Lamniform shark from the waters adjacent to Taiwan. *Journal of the Fisheries Society of Taiwan*. **2009**, *36(4)*, 303–311.
14. Tanaka, T., Noguchi, F., and Tanaka, S. Dentition of a male megamouth shark, *Megachasma pelagios* from Suruga Bay, Japan, with a comparison of the fossil shark teeth from Chile. In: Report of Japanese Society for Elasmobranch Studies. **2004**, *40*, 31–37.
15. White, W.T.; Fahmi, M.A.; Sumadhiharga, K. A juvenile megamouth shark *Megachasma pelagios* (Lamniformes: Megachasmidae) from Northern Sumatra, Indonesia. *Raffles Bull. Zool.* **2004**, *52*, 603–607.
16. Iida, M. Catch of megamouth shark by set net in Sagami Bay. In: Report of Japanese Society for Elasmobranch Studies. **2004**, *40*, 38–40.
17. Summary of Megamouth Sharks. Available online: http://elasmollet.org/Mp/Mplist.html (accessed on 1 September 2021).
18. Castillo-Géniz, J. L., Ocampo-Torres, A. I., Shimada, K., Rigsby, C. K., and Nicholas, A. C. Juvenile megamouth shark, *Megachasma pelagios*, caught off the Pacific coast of Mexico, and its significance to chondrichthyan diversity in Mexico. *Cienc. Mar.* **2012**, *38(2)*, 467–474.
19. Sharkmans-world. Available online: https://sharkmans-world.blogspot.com/ (accessed on 1 September 2021).
20. Sawamoto, S.; Matsumoto, R. Stomach contents of a megamouth shark Megachasma pelagios from the Kuroshio Extension: Evidence for feeding on a euphausiid swarm. *Plankton Benthos Res.* **2012**, *7*, 203–206, https://doi.org/10.3800/pbr.7.203.
21. de Moura, J. F., Merico, A., Montone, R. C., Silva, J., Seixas, T. G., de Oliveira Godoy, J. M., Pierre, T. D., Hauser-Davis, R. A., Di Beneditto, A. P. M., Reis, E. C., Tavares, D. C., Lemos, L. S., and Siciliano, S. Assessment of trace elements, POPs, 210Po and stable isotopes (15N and 13C) in a rare filter-feeding shark: The megamouth. *Mar. Pollut. Bull*. **2015**, *95(1)*, 402–406.
22. Senou, H., Taru, H., and Tanaka, S. Record of a megamouth shark, *Megachasma pelagios* (Elasmobranchii: Megachasmidae), from Sagami Bay.In: *Report of Japanese Society for Elasmobranch Studies*. **2012**, *48*, 21–27
23. Asahi Shimbun. Available online: https://www.asahi.com/ (accessed on 1st September 2021).
24. Misawa, R., Wada, J., Kitadani, Y., Nishida, K., Kai, Y., Mizumachi, K., Endo, H. A checklist of sharks based on voucher specimens

and photographs from Kochi Prefecture (southern Shikoku Island, Japan). In: *Report of Japanese Society for Elasmobranch Studies*. **2019**, *55*, 31–54

25. Elusive megamouth shark snared in Mexico. Available online: http://weareseaborn.blogspot.com/2011/09/elusive-megamouth-shark-snared-in.html (accessed on 1st September 2021).
26. Tomita, T.; Tanaka, S.; Sato, K.; Nakaya, K. Pectoral Fin of the Megamouth Shark: Skeletal and Muscular Systems, Skin Histology, and Functional Morphology. *PLoS ONE* **2014**, *9*, e86205, https://doi.org/10.1371/journal.pone.0086205.
27. Martínez-Ortiz, J.; Mendoza-Intriago, D.; Tigrero-Gonzalez, W.; Flores-Rivera, G.; López-Parraga, R. New records of megamouth shark, *Megachasma pelagios* off Ecuador, Eastern Pacific Ocean. *Cienc. Pesq.* **2017**, *25*, 27–30.
28. Fernando, D.; Perera, N.; Ebert, D.A. First record of the megamouth shark, *Megachasma pelagios*, (Chondrichthyes: Lamniformes: Megachasmidae) from Sri Lanka, Northern Indian Ocean. *Mar. Biodivers. Rec.* **2015**, *8*, e75, https://doi.org/10.1017/s1755267215000512.
29. Chang, C.-H.; Shao, K.-T.; Lin, Y.-S.; Chiang, W.-C.; Jang-Liaw, N.-H. Complete mitochondrial genome of the megamouth shark *Megachasma pelagios* (Chondrichthyes, Megachasmidae). *Mitochondrial DNA* **2013**, *25*, 185–187, https://doi.org/10.3109/19401736.2013.792068.
30. Tanaka, S., Horie, T., and Yuki, Y. Occurrence and catch records of megamouth shark, Megachasma pelagios in Japan from 2013 to 2014. In: *Report of Japanese Society for Elasmobranch Studies*. **2014**, *50*, 35–39
31. Senou, H. The largest megamouth shark in Japan collected from Sagami Bay. In: *Report of Japanese Society for Elasmobranch Studies*. **2013**, *49*, 18–20.
32. Fujii, M. Bycatch of megamouth shark by a set net in the east coast of Izu. In: *Report of Japanese Society for Elasmobranch Studies*. **2015**, *51*, 21–23.
33. Chukyo TV. Available online: https://www.ctv.co.jp/indexmenu.html (accessed on 1st September 2021).
34. Kelez, S.; Napuri, R.M.; Pfennig, A.M.; Martinez, O.C.; Carrasco, A.T. First reports of Megamouth Shark, *Megachasma pelagios* Taylor, Compagno & Struhsaker, 1983 (Lamniformes, Megachasmidae), in Peru. *Check List*. **2020**, *16*, 1361–1367, https://doi.org/10.15560/16.5.1361.
35. Rodriguez-Ferrer, G., Wetherbee, B. M., Schärer, M., Lilyestrom, C., Zegarra, J. P., and Shivji, M. First record of the megamouth shark, *Megachasma pelagios*, (family Megachasmidae) in the tropical western North Atlantic Ocean. Marine Biodiversity Records. **2017**, *10(1)*, 1-4.
36. Duchatelet, L.; Moris, V.C.; Tomita, T.; Mahillon, J.; Sato, K.; Behets, C.; Mallefet, J. The megamouth shark, *Megachasma pelagios*, is not a luminous species. *PLoS ONE* **2020**, *15*, e0242196, https://doi.org/10.1371/journal.pone.0242196.
37. Acuña-Perales, N.; Córdova-Zavaleta, F.; Alfaro-Shigueto, J.; Mangel, J.C. First records of the megamouth shark *Megachasma pelagios* (Taylor, Compagno & Struhsaker, 1983) as bycatch in Peruvian small-scale net fisheries. *Mar. Biodivers. Rec.* **2021**, *14*, 1–7, https://doi.org/10.1186/s41200-020-00198-x.
